# Over-Expression of Porcine Myostatin Missense Mutant Leads to A Gender Difference in Skeletal Muscle Growth between Transgenic Male and Female Mice

**DOI:** 10.3390/ijms160820020

**Published:** 2015-08-24

**Authors:** Dezun Ma, Pengfei Gao, Lili Qian, Qingqing Wang, Chunbo Cai, Shengwang Jiang, Gaojun Xiao, Wentao Cui

**Affiliations:** 1 State Key Laboratory for Animal Nutrition, Institute of Animal Science, Chinese Academy of Agricultural Sciences, Beijing 100193, China; E-Mails: madezun@126.com (D.M.); pengfei.gao@yahoo.com (P.G.); qingqingwang893@yahoo.com (Q.W.); jshengwang@yahoo.com (S.J.); gaojunx@yahoo.com (G.X.); 2 College of Biological Sciences, China Agricultural University, Beijing 100193, China; E-Mails: lili.qian@yahoo.com (L.Q.); caichunbobo@126.com (C.C.)

**Keywords:** porcine myostatin, transgenic mice, muscle growth, androgen receptor 45

## Abstract

Myostatin, a transforming growth factor-β family member, is a negative regulator of skeletal muscle development and growth. Piedmontese cattle breeds have a missense mutation, which results in a cysteine to tyrosine substitution in the mature myostatin protein (C313Y). This loss-of-function mutation in myostatin results in a double-muscled phenotype in cattle. Myostatin propeptide is an inhibitor of myostatin activity and is considered a potential agent to stimulate muscle growth in livestock. In this study, we generated transgenic mice overexpressing porcine myostatin missense mutant (pmMS), C313Y, and wild-type porcine myostatin propeptide (ppMS), respectively, to examine their effects on muscle growth in mice. Enhanced muscle growth was observed in both pmMS and ppMS transgenic female mice and also in ppMS transgenic male mice. However, there was no enhanced muscle growth observed in pmMS transgenic male mice. To explore why there is such a big difference in muscle growth between pmMS and ppMS transgenic male mice, the expression level of androgen receptor (AR) mutant AR45 was measured by Western blot. Results indicated that AR45 expression significantly increased in pmMS transgenic male mice while it decreased dramatically in ppMS transgenic male mice. Our data demonstrate that both pmMS and ppMS act as myostatin inhibitors in the regulation of muscle growth, but the effect of pmMS in male mice is reversed by an increased AR45 expression. These results provide useful insight and basic theory to future studies on improving pork quality by genetically manipulating myostatin expression or by regulating myostatin activity.

## 1. Introduction

Myostatin is well-known as a growth and differentiation factor 8 (GDF-8) that acts as a negative regulator of skeletal muscle growth. Myostatin is secreted as a precursor protein which is cleaved by multiple steps of furin-type proteases to generate an N-terminal propeptide and a C-terminal growth factor dimer, the biologically active part [1]. The N-terminal propeptide remains bound to the C-terminal dimer to form the inactive latent complex [2,3]. Mature myostatin is liberated from the latent myostatin complex by bone morphogenetic protein 1/tolloid proteases. The myostatin gene is extraordinarily well conserved among different species and the amino acid sequences of the mature and active protein from humans, mice, rats, pigs, and chickens are identical [4]. The skeletal muscle weight of GDF-8 (myostatin) null mice is twice as much as wild-type mice [5]. Enhanced muscle growth was observed in transgenic mice expressing myostatin propeptide [6,7]. Recently, transgenic mice expressing wild-type porcine myostatin propeptide were also reported to have enhanced muscle growth [8].

Several studies have demonstrated that the naturally occurring loss-of-function mutation in the bovine myostatin gene in the Piedmontese breed of cattle (showing a double-muscled phenotype) is a missense mutation, which results in a cysteine-to-tyrosine substitution in the mature myostatin protein (C313Y) [9,10,11,12].

In addition to myostatin, androgens also play a key role in skeletal muscle growth. Androgens exert their effects by binding to androgen receptors (AR), a ligand-inducible transcription factor [13]. On one hand, androgens stimulate muscle growth and increase the size of myofibers. On the other hand, AR can stimulate the expression of myostatin that, in turn, could inhibit muscle growth [14]. AR45, a naturally occurring variant of androgen receptor, is composed of the AR DNA-binding domain, hinge region, and ligand-binding domain, preceded by a novel seven-amino-acid-long N-terminal extension. It is reported that AR45 is mainly expressed in the heart and skeletal muscles [15]. AR45 has been shown to inhibit AR function by forming AR-AR45 heterodimers. Thus, it seems that androgens’ regulatory role in muscle growth may be, in part, related to the regulation of myostatin expression by androgens.

The pig is a key livestock animal and is a major source of meat. It is thus very important to improve the quality (such as lean yield and fat content) of pork by either genetically engineering the structure of myostatin or by intervention of myostatin’s function with specific inhibitors.

In this study, we generated two types of transgenic mice overexpressing porcine missense mutation myostatin (pmMS) or wild-type porcine propeptide myostatin (ppMS) to compare their effects on myostatin-mediated muscle growth and the AR signaling pathway. Data obtained in this study provide useful insight and basic theory to future studies on improving pork quality by genetically manipulating myostatin expression or by regulating myostatin activity.

## 2. Results

### 2.1. Generation Transgenic Mice

**Figure 1:**
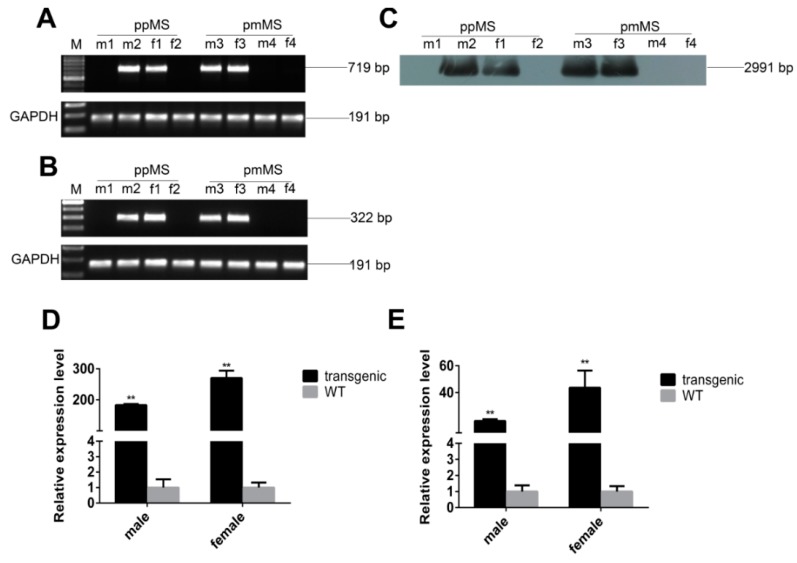
Identification and expression of pmMS and ppMS transgenes in mice. (**A**) Identification of transgenic mice by PCR; (**B**) Identification of transgenic mice by RT-PCR; (**C**) Identification of transgenic mice by Southern blot; (**D**) The mRNA expression level of exogenous pmMS and endogenous myostatin and (**E**) The mRNA expression level of exogenous ppMS and endogenous myostatin. There were five transgenic mice and six WT (wild type) mice in each group. M, marker; ppMS, wild-type porcine myostatin propeptide; pmMS, porcine myostatin missense mutant; m1, WT male mice; m2, transgenic male mice expressing ppMS; f1, transgenic female mice expressing ppMS; f2, WT female mice; m3, transgenic male mice expressing pmMS; m4, WT male mice; f3, transgenic female mice expressing pmMS; f4, WT female mice. ** *p* < 0.01.

Both types of transgenic mice were confirmed by PCR and Southern blot (Figure 1A,C). Since there is a difference in gene sequence between C57 BL/6 mice and porcine myostatin, we used reverse transcription PCR by designing primers that only amplify exogenous porcine myostatin to identify the genotype of transgenic mice at the RNA level (Figure 1B). Based on the real-time quantitative PCR (RT-PCR) results, the mRNA expression level of total myostatin, which contains both exogenous porcine and murine endogenous myostatin, was much higher in transgenic mice than in wild-type (WT) mice (Figure 1D,E). Our data confirm that both types of transgenic mice were successfully generated.

### 2.2. Phenotype and Characterization of Transgenic Mice

**Figure 2:**
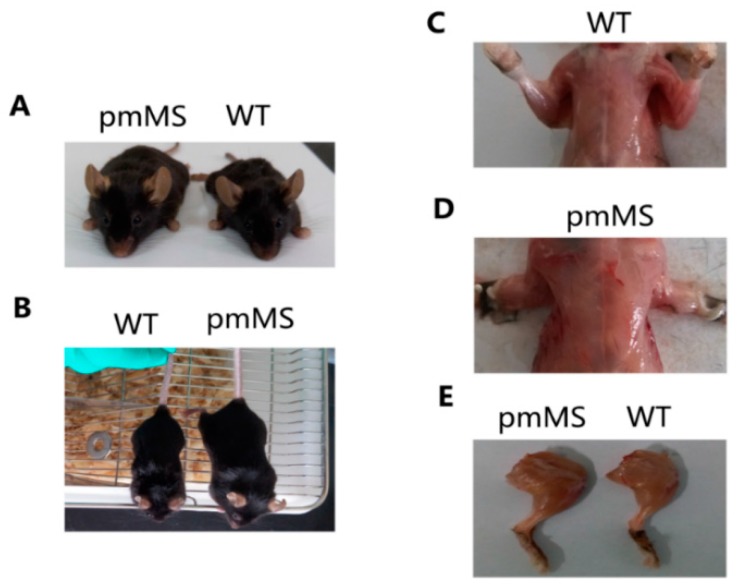
Increased body mass and skeletal muscle weight of pmMS transgenic female mice compared with wild-type mice. (**A**,**B**): Increased body mass in pmMS transgenic female mice, pictures taken from different angles; (**C**–**E**): Increased muscling in pmMS transgenic female mice. WT, wild type.

To determine whether the expression of pmMS or ppMS in transgenic mice has any effect on muscle growth, we dissected the skeletal muscle of the hind limb that contains the gastrocnemius, rectusfemoris, tibialis anterior (TA), and pectoralis, and measured each muscle’s weight, respectively. The average body weight increased by 6.54% and 9.48% in pmMS and ppMS transgenic female mice, respectively, compared to WT mice (Table 1). The average weight of each individual muscle increased by 17.46%, 32.36%, 35.75%, and 22.16% in pmMS transgenic female mice, and 32.06%, 58.84%, 38.41%, and 41.79% in ppMS transgenic female mice, respectively, compared to WT mice (Table 1). However, the muscle mass weight did not change in pmMS transgenic male mice, while it increased by 31.74%, 52.71%, 49.64%, and 37.61% in ppMS transgenic male mice compared with WT mice (Table 1). Figure 2A,B show pictures of a representative WT female mouse and a representative pmMS female mouse. Figure 2C–E show the hind limb and pectoralis from a pmMS transgenic female mouse and a WT female mouse, respectively. It is clear that the transgenic pmMS female mouse looks more muscular than the WT mouse. The weight of viscus did not change in both types of transgenic mice compared with WT mice (data not shown).

**Table 1:** Body and muscle weights (g) of pmMS and ppMS transgenic mice.

Offspring	Sex	*n*	Body Weight	Gastrocnemius	Rectusfemoris	TA	Pectoralis
pmMS	male	5	35.3 ± 3.2	0.1936 ± 0.0114	0.1717 ± 0.0385	0.0708 ± 0.0074	0.3325 ± 0.0396
ppMS	male	5	36.7 ± 2.0 ^*^	0.2536 ± 0.0433 ^**^	0.2286 ± 0.0174 ^**^	0.1040 ± 0.0140 ^**^	0.4427 ± 0.0803 ^**^
controls	male	8	34.2 ± 2.0	0.1925 ± 0.0217	0.1497 ± 0.0282	0.0695 ± 0.0114	0.3217 ± 0.0477
pmMS	female	5	32.6 ± 1.4 ^*^	0.1843 ± 0.0091 ^**^	0.1714 ± 0.0253 ^*^	0.0767 ± 0.0065 ^**^	0.2833 ± 0.0145 ^**^
ppMS	female	5	33.5 ± 2.0 ^*^	0.2072 ± 0.0286 ^**^	0.2057 ± 0.0342 ^**^	0.0782 ± 0.0156 ^*^	0.3288 ± 0.0547 ^**^
controls	female	7	30.6 ± 1.3	0.1569 ± 0.0109	0.1295 ± 0.0265	0.0565 ± 0.0080	0.2319 ± 0.0273

ppMS, wild-type porcine myostatin propeptide; pmMS, porcine myostatin missense mutant; TA, anterior tibialis; * *p* < 0.05; ** *p* < 0.01.

**Figure 3:**
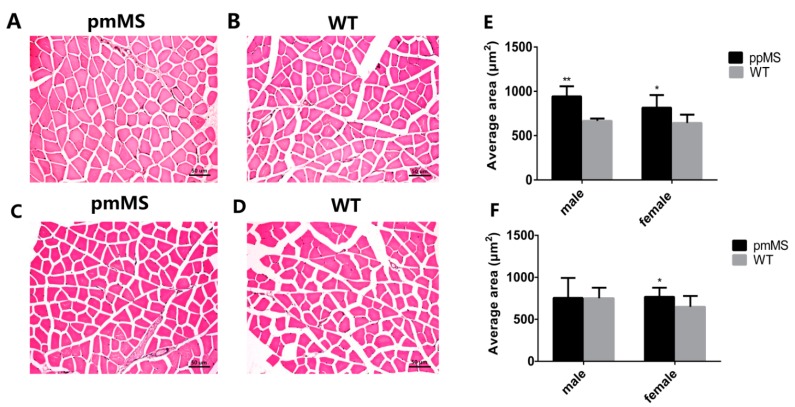
Analysis of myofiber cell size of gastrocnemius from transgenic and WT mice at the age of 30 weeks. (**A**) Muscle cross-sections from pmMS transgenic female mice; (**B**) Muscle cross-sections from WT female mice; (**C**) Muscle cross-sections from pmMS transgenic male mice; (**D**) Muscle cross-sections from WT male mice; (**E**) Comparsion of average muscle size between ppMS transgenic mice (male: *n* = 5; female: *n* = 5) and WT mice (male: *n* = 8; female: *n* = 6) and (**F**) Comparsion of average muscle size between pmMS transgenic mice (male: *n* = 5; female: *n* = 5) and WT mice (male: *n* = 8; female: *n* = 6). Scale bars = 50 μm; * *p* < 0.05,** *p* < 0.01.

Next, we measured the myofiber size in the gastrocnemius by hematoxylin and eosin and the results show that the mean gastrocnemius fiber size increased by 18.27% and 26.77%, respectively, in pmMS and ppMS transgenic female mice compared with WT mice (Figure 3A,B,E,F). However, compared with WT male mice, the mean gastrocnemius fiber size in pmMS transgenic male mice did not change, but it increased 41.39% in ppMS transgenic male mice (Figure 3C–F).

**Figure 4:**
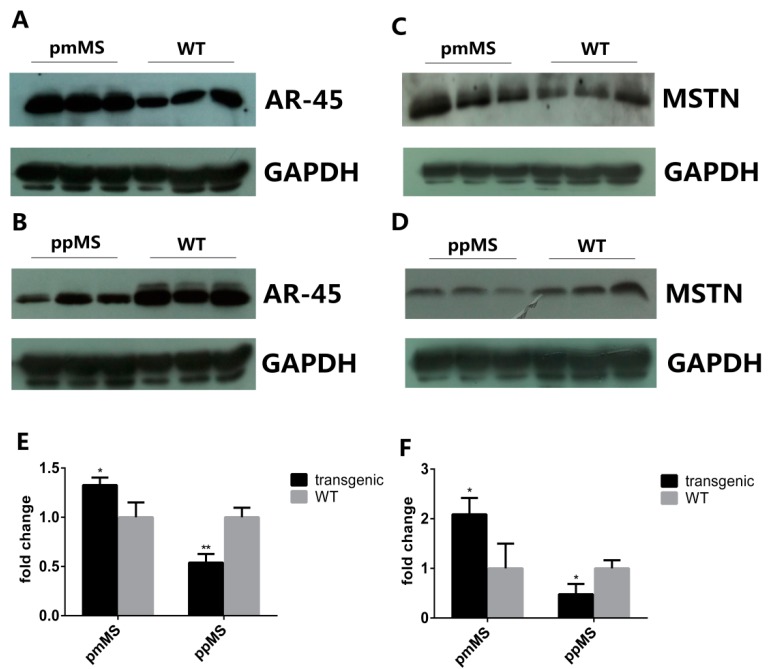
Protein expression level of AR45 and myostatin in the gastrocnemius of transgenic male mice and WT male mice by Western blot. (**A**) Protein expression level of AR45 (67 kDa) in pmMS transgenic male and WT male mice; (**B**) Protein expression level of AR45 (67 kDa) in ppMS transgenic male and WT male mice; (**C**) Protein expression level of myostatin (26 kDa) in pmMS transgenic male and WT male mice; (**D**) Protein expression level of myostatin (26 kDa) in ppMS transgenic male and WT male mice. In panels (**A**–**D**), each lane contains a sample from one individual mouse; (**E**) Quantitation of AR45 protein band intensity by densitometry using image J software and (**F**) Quantitation of myostatin protein band intensity by densitometry using image J software (NIH, Bethesda, MD, USA). GAPDH was used as an internal reference. MSTN, myostatin; * *p* < 0.05, ** *p* < 0.01.

**Figure 5:**
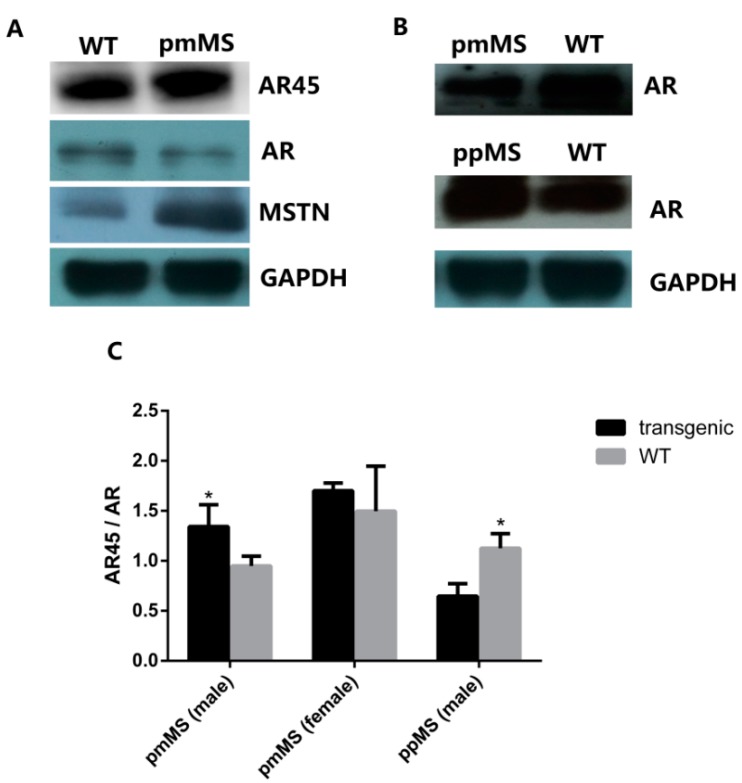
Protein expression level of AR45, AR, and myostatin in the gastrocnemius of two lines of transgenic mice and WT male mice by Western blot. (**A**) Protein expression of AR (110 kDa), AR45 (67 kDa), and myostatin (26 kDa) in pmMS transgenic female and WT female mice; (**B**) Protein expression of AR (110 kDa) in pmMS and ppMS transgenic male and WT male mice and (**C**) AR45/AR ratios calculated by densitometry using image J software. AR, androgen receptor. * *p* <0.05.

To explore why there is such a huge difference in muscle growth and fiber size between pmMS and ppMS transgenic male mice, the AR45 protein level was analyzed by Western blot. The intensity of the AR45 protein band in each genotype of male mice was measured by densitometry using Image J 1.48u (NIH, Bethesda, MD, USA). Results indicated that compared with WT male mice, the level of AR45 protein increased 32.65% in pmMS transgenic male mice but decreased 46.07% in ppMS transgenic male mice (Figure 4A,B,E). On the other hand, the AR45 level is only slightly higher in pmMS female mice than in wild-type female mice (Figure 5A); the difference in the AR45 level between pmMS female mice and wild-type female mice is not significant.

Results of the Western blot for mature myostatin (MSTN) indicated that the level of total mature myostatin peptides that include endogenous murine MSTN and exogenous C313Y mutated porcine MSTN (molecular weight of homodimer of mature myostatin peptide is about 26 kDa under non-reducing condition) is 108.81% higher in pmMS transgenic male mice and 52.03% lower in ppMS transgenic male mice than in WT male mice, respectively (Figure 4C,D,F). Please note that in pmMS transgenic mice, since both endogenous murine MSTN and exogenous C313Y mutated porcine MSTN are expressed, and the anti-MSTN antibody used in Western blot detection is specific for total mature MSTN, the native murine and mutated porcine MSTN cannot be distinguished. Therefore, as expected and shown in Figure 4C and Figure 5A, pmMS transgenic mice contain much higher total MSTN levels than the corresponding wild-type mice. We also performed a Western blot with crude extracts from gastrocnemius of a representative pmMS male mouse and and a WT male mouse under non-reduced condition and our results show that pro-myostatin and mature myostastin protein bands were detected (Figure S1).

Theoretically speaking, as has been demonstrated in Piedmontese cattle [12], it is expected that exogenous C313Y mutated MSTN can inhibit active endogenous murine MSTN function and thus can enhance muscle growth and lead to an increase in muscle mass. However, enhanced muscle growth was only observed in pmMS female mice, but not in pmMS male mice. Based on results of total mature MSTN and AR45 expression levels in pmMS male and female mice, along with AR45 data from ppMS male mice, we speculate that the increased level of C313Y mutated MSTN and its binding to the MSTN receptor led to a significantly increased AR45 expression level through a different signaling pathway. However, further investigation is required to confirm this idea.

We also performed an AR Western blot (Figure 5). As expected, female mice express a lower level of AR than male mice. The AR level decreased in both pmMS female and male mice, but the decrease was not significant for pmMS female mice, while it was significant for pmMS male mice (Figure 5A,B). It is interesting to note that the AR level increased in ppMS male mice. The calculation of AR45/AR ratios from density data of AR and AR45 bands showed there is not a significant (*p* > 0.05) difference between pmMS female mice and wild-type female mice, but there is a significant difference between pmMS male mice and wild-type male mice (*p* < 0.05) (Figure 5C). These data further suggest that the increased level in total MSTN led to an increase in the AR45 level and a decrease in the AR level in pmMS male mice. The non-significant change in the AR45/AR ratio in pmMS female mice may explain why there is a MSTN mutant-induced increase in muscle mass.

Additionally, as a comparison, we performed an AR Western blot for samples from ppMS male mice. Data indicated that the AR level increased in ppMS male mice compared to wild-type male mice, and a significant difference was seen in the AR45/AR ratio between ppMS male mice and wild-type male mice (Figure 5B,C). The result of the AR45/AR ratio in ppMS male mice is just the opposite to what was observed in pmMS male mice, and it provides additional evidence to support our speculation that higher-mutated MSTN levels in pmMS male mice result in higher levels of AR45, which in turn may reverse the increase in muscle mass induced by mutated MSTN as observed in female pmMS mice.

## 3. Discussion

Our results in this study indicate that there is a different pattern of changes in muscle mass between pmMS transgenic male and female mice. For example, an increased muscle mass was observed in female pmMS transgenic mice but no change in male pmMS transgenic mice was noticed when compared to WT mice. We hypothesize that this big sexual difference between female and male mice could be due to the different levels of secreted androgens. This hypothesis was based on the fact that, on one hand, androgens can activate muscle androgen receptor (AR) to regulate muscle mass, and on the other hand, they can also induce myostatin signaling (by increasing the myostatin expression level) to restrain their own anabolic actions [13,14]. In addition, one of the AR mutants, AR45, is mainly found in skeletal muscle and the heart, and it plays an inhibitory role in AR functions [15]. Results from the Western blot showed that the AR45 expression level in muscle from male pmMS transgenic mice is significantly higher than in muscle from WT male mice. On the other hand, the AR45 expression level in muscle from male ppMS transgenic mice is dramatically lower. Based on these different changes in the AR45 level and the fact that pmMS expression can stimulate muscle growth in female transgenic mice, we believe that the pmMS-enhanced effect on muscle growth was reversed by the increased AR45 expression level, which is known to inhibit AR functions. Therefore, the balance between the AR45 and myostatin signaling pathways results in no net change in muscle mass in male pmMS transgenic mice. Contrary to what was observed in male pmMS transgenic mice, there is a significant increase in muscle mass and myofiber size in male ppMS transgenic mice. This is consistent with the fact that, on one hand, ppMS inhibits myostatin activity and thus enhances muscle growth and, on the other hand, the ppMS-induced decrease in the AR45 expression level also contributes to muscle growth in male ppMS transgenic mice.

To explain why there is such a big difference in AR45 expression levels between ppMS and pmMS transgenic male mice, we hypothesized that the different levels of mature myostatin may be the key reason. It is notable that in pmMS transgenic mice, a full length of myostatin is inserted and thus a higher level of total mature myostatin (including endogenous murine MSTN and exogenous C313Y mutated porcine MSTN) is produced, which, in turn, may increase the AR45 expression level; however, in ppMS transgenic mice, only the propeptide gene is inserted and, as a result, the propeptide will bind to mature myostatin and may lead to a decrease in the level of mature myostatin. Indeed, Western blot results on mature myostatin are consistent with the above hypothesis. Thus, we believe that the level of mature myostatin in transgenic male mice has a positive relationship with AR45 expression.

To our knowledge, there has been no report on sexually differential responses between female and male Piedmontese cattle. One of the key reasons why we observed sexually different responses in pmMS transgenic mice is that the endogenous murine MSTN and the exogenous, inactive C313Y MSTN co-exist, with the latter being the dominant form. In Piedmontese cattle, C313Y MSTN is the only form of MSTN. Additionally, cattle and mice are very different species. Taken together, this could explain why there is a sexually different response in pmMS mice while there is such difference observed in Piedmontese cattle.

## 4. Experimental Section

### 4.1. Generation of pmMS and ppMS Transgenic Mice

Two plasmids were constructed by inserting porcine missense mutation myostatin (C313Y) and wild-type propeptide gene sequence into “Sleeping Beauty” transposon (pT2-HB) carrier that was digested with Not I restriction enzyme. The pmMS and ppMS gene sequences were amplified, to which Not I restriction enzyme sites were added at each end. Then, pT2-HB was digested with Not I restriction endonuclease and connected with the pmMS and ppMS gene sequence, respectively, by T4 ligase. These two DNA recombinant plasmids were designated as SB-MSTN313 and SB-MSTNpro, respectively. The linearized SB-MSTN313 and SB-MSTNpro was microinjected into the male pronuclear of fertilized C57BL/6J mouse eggs with SB100 transposase, respectively, to generate transgenic mice.

Genomic DNA was extracted from tail of each transgenic mouse and detected by PCR. The primes, which amplify the sequence of SV40 and MLC1 enhancer sequence, are as follows: 5′-CACTGCATTCTAGTTGTGGTTTGT-3′ and 5′-AAGCATGATGTCTGTGCGGT-3′. The PCR condition was 95 °C for 5 min, 35 cycles including 30 s at 95 °C, 30 s at 64 °C, 40 s at 72 °C, and 72 °C for 10 min. The primers for amplification of mouse GADPH were as follows: 5′-ACCCAGAAGACTGTGGATGG-3′ and 5′-CACATTGGGGGTAGGAACAC-3′. All PCR products were sequenced to confirm the transgene identities.

Detection of transgenic mice by reverse-transcription (RT-PCR): The primers designed for only amplifying the exogenous porcine pmMS and ppMS sequences were as follows: 5′-AGAACAGCGAGCAAAAGGAAA-3′ and 5′-TCCACTTGCATTAGAAGATCAGA-3′. PCR was performed under the following conditions: 95 °C for 5 min, 35 cycles including 30 s at 95 °C, 30 s at 60 °C, 20 s at 72 °C, and 72 °C for 10 min.

The two different types of transgenic mice were also confirmed by Southern blot. Genomic DNA (15 µg) was digested by SpeI and electrophoresed on 0.8% gels. DNA was then transferred to nylon membrane (GE Healthcare, Pittsburgh, PA, USA) and hybridized with the probe that was labeled by using DIG High Prime DNA Labeling and Detection Starter kit I (Roche, Mannheim, Germany). The probe sequences were as follows: 5′-CACTGCATTCTAGTTGTGGTTTGT-3′ and 5′-AAGCATGATG TCTGTGCGGT-3′.

Heterozygous F1 transgenic mice were generated by breeding transgenic C57BL/6J founder (F0) male mouse with wild-type C57BL/6J female mouse. The F1 transgenic mice, which come from the same transgenic male parent (half siblings), were used in this study at the age of 30 weeks.

### 4.2. Real-Time Quantitative PCR

The real-time quantitative PCR was used to measure mRNA levels of murine endogenous, porcine exogenous myostatin in transgenic and non-transgenic (wild type) mice. Total RNA of gastrocnemius was extracted using Trizol (Invitrogen, Shanghai, China) according to manufacturer’s protocol. The cDNA of total RNA was synthesized by using the First Strand cDNA Synthesis kit (Fermentas, Beijing, China), and the real-time quantitative PCR was performed by using SYBR Green kit (Applied Biosystems, Beijing, China) and the ABI-7500 Real-Time PCR system (Applied Biosystems). The method was basically the same as the previously described protocol [16]. Specific primers, which amplify the exogenous and endogenous myostatin, were as follows: 5′-agtgatggctccttggaaga-3′ and 5′-tgtaggagtcttgacgggtc-3′. All data were calculated by the 2^−∆∆*C*t^ method.

### 4.3. Western Blot Analysis

Protein samples were extracted from gastrocnemius of transgenic and wild-type (WT) male mice, separated by SDS-PAGE, and then electrotransferred onto a PVDF membrane, followed by overnight blocking with 5% fat-free milk. For AR45 detection, the membrane was incubated with a rat anti-AR45 antibody (Abcam, Shanghai, China) for 2 h at room temperature, followed by incubation rabbit with anti-rat antibody for 1 h at room temperature. For Western blot of mature myostatin peptide, SDS-PAGE was performed under non-reducing condition. For SDS-PAGE under non-reducing condition, samples were heated at 100 °C for 5 min in sample buffer without any reducing reagent such as β-mercaptoethanol, and SDS gel was then run using a running buffer containing no reduced reagent such as β-mercaptoethanol. Under this non-reduced condition, disulfide bonds in proteins are not broken. For example, pro-myostatins (which contains signal peptide, propeptide) will be in their dimer forms (with a molecular weight of about 90 kDa for pro-myostatin and about 26 kDa for mature MSTN). The primary antibody is rabbit anti-myostatin (Abcam, catalog number ab98337) that recognizes C-terminus of myostatin and the secondary antibody is goat anti-rabbit antibody (CST). GAPDH served as an internal reference. Each blot was detected using SuperSignal West Pico Chemiluminescent Substrate (Thermo Scientific, Shanghai, China), and the intensity of protein bands was measured by image J software 1.48u.

### 4.4. Animal Care and Histological Analysis

All experimental mice were of C57BL/6J strain. They were maintained under the following conditions: temperature of 25 ± 1 °C, relative humidity of 70% ± 4%, automatic light control, rearing density less than or equal to 4 per cage. Mice were provided food and water *ad libitum*. All animal experiments were approved by the Institutional Animal Care and Use Committee of Chinese Academy of Agricultural Sciences.

All mice were humanely euthanized, and muscles containing gastrocnemius, rectusfemoris, tibialis anterior (TA), and pectoralis were dissected and weighed as previously described [17].

### 4.5. Measurement of Muscle Fiber Size

The gastrocnemius was dissected at the time when mice were euthanized, fixed in 4% paraformaldehyde, and embedded in paraffin. Muscle sections were stained with hematoxylin and eosin, and pictures were taken from four random fields at 400× magnification. Image J software was used to calculate the muscle cross-sectional area using the method as previously described [8].

### 4.6. Statistical Analysis

All statistical analyses were performed by SAS program 9.2 software (SAS Institute Inc, North carolina, NC, USA). Student’s *t*-test was used to analyze differences between the two groups. Values of *p* < 0.05 and *p* < 0.01 were labeled * and **, respectively.

## 5. Conclusions

In summary, we have successfully generated, for the first time, pmMS transgenic mice, and made a comparative study with ppMS transgenic mice. The enhanced effects of these two myostatin inhibitors on muscle development and growth were demonstrated in these transgenic mice. However, these two myostatin inhibitors have very different effects on muscle growth and AR45 expression in male mice, suggesting that myostatin inhibitors regulate skeletal muscle growth differently via their different myostatin-mediated effects on the androgen signaling pathway.

## Supplementary Materials

Supplementary materials can be found at http://www.mdpi.com/1422-0067/16/08/20020/s1.
