# Supplementary material for: Over-Expression of Porcine Myostatin Missense Mutant Leads to A Gender Difference in Skeletal Muscle Growth between Transgenic Male and Female Mice
Source: Int J Mol Sci. 2015 Aug 24;16(8):20020–32. doi: 10.3390/ijms160820020 (PMC4581338; doi:10.3390/ijms160820020)
Supplement: Supplementary file 1 [file ijms-16-20020-s001.pdf]

Supplementary Information

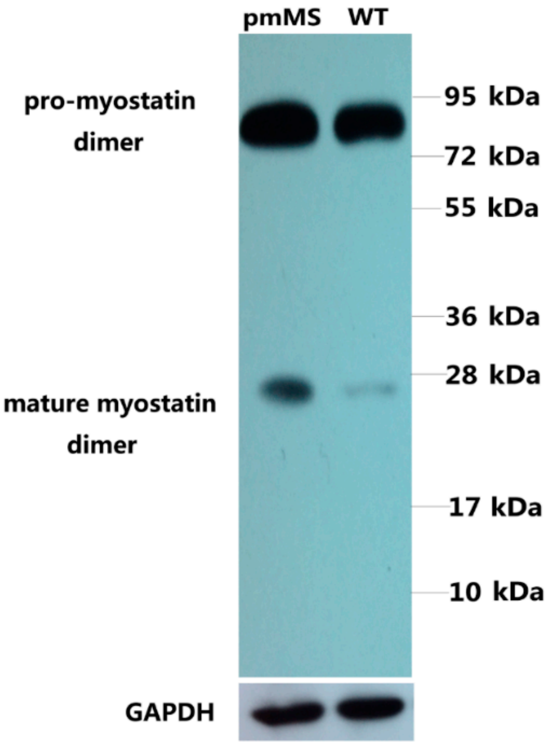

Figure S1. Western blot of extracts isolated from gastrocnemius of a representative pmMS (transgenic mice expressing porcine missense mutation myostatin) male mouse and a WT (wild type) male mouse. SDS-PAGE was performed under non-reduced condition as decribed in Experimental Section. *Pro-myostatin* refers to full-length myostatin containing signal peptide, propeptide and mature myostatin. WT, wild type.
